# Small RNAs in the peripheral blood discriminate metastasized from non-metastasized seminoma

**DOI:** 10.1186/1476-4598-13-47

**Published:** 2014-03-06

**Authors:** Christian G Ruf, Daniela Dinger, Matthias Port, Hans-Ulrich Schmelz, Walter Wagner, Cord Matthies, Bertram Müller-Myhsok, Viktor Meineke, Michael Abend

**Affiliations:** 1 Department of Urology, Federal Armed Forces Hospital, 22049 Hamburg, Germany; 2 Bundeswehr Institute of Radiobiology, 80937 Munich, Germany; 3 Department of Urology, Federal Armed Forces Central Hospital, 56072 Koblenz, Germany; 4 Department of Hematology, Hemostasis Oncology and Stem Cell Transplantation, MHH, Hannover, Germany; 5 Max Planck Institute of Psychiatry, Munich, Germany

**Keywords:** Testis tumour, Gene expression, Small RNA, MicroRNA, Metastasized seminoma, Next generation sequencing, Risk factor, Tumour marker, Blood

## Abstract

**Background:**

We aimed to better discriminate metastasized (lymphogen/occult/both combined) from non-metastasized seminoma based on post-transcriptional changes examined in the peripheral blood.

**Methods:**

Total RNAs including small RNAs were isolated from the peripheral blood of patients suffering from metastasized testicular tumours (lymphogen, n = 5, clinical stage IIb/c; occult, n = 5, clinical stage I) and non-metastasized patients (n = 5, clinical stage I). Small RNA next generation sequencing (SOLID, Life Technologies) was employed to examine post-transcriptional changes. We searched for small RNAs showing at least 50 reads and a significant ≥ 2-fold difference using peripheral blood small RNAs of non-metastasized tumours as the reference group. Candidate small RNAs were examined in univariate logistic regression analysis and combinations of two small RNAs were further examined using support vector machines.

**Results:**

On average 1.3x10^7^, 1.2x10^7^ and 1.2x10^7^ small RNA reads were detectable in non-metastasized, lymphogen and occult metastasized seminoma, respectively of which 73-76% remained after trimming. From these between 80-82% represented annotated reads and 7.2-7.8% (1.6-1.7x10^4^) were annotated small RNA tags. Of them 137 small RNAs showed > 50 reads and a ≥ two-fold difference to the reference. In univariate analysis we detected 33-35 different small RNAs which significantly discriminated lymphogen/occult/combined metastasized from non-metastasized seminoma and among these different comparisons it were the same small RNAs in 44-79%. Many combinations of two of these small RNAs completely discriminated metastasized from non-metastasized seminoma irrespective of the metastasis subtype.

**Conclusions:**

Metastasized (either lymphogen or occult) seminoma can be completely discriminated from non-metastasized seminoma with a combination of two small RNAs measured in the peripheral blood.

## Background

Testicular tumor, as the most common tumor in young men, is associated with a 5 year survival rate close to 100% in early stages. Pure seminoma are the most frequent histological subtype (55%) nowadays and more than 70% of patients present without visible metastasis at primary staging [1]. Gold standard for primary staging is computed tomography (CT) of the chest, abdomen and pelvis to detect metastases. In truly non-metastasized clinical stage I (cS I) patients are cured by orchidectomy alone, but despite modern staging and classification procedures up to 30% of cS I seminoma patients’ bear occult metastasis in primary staging and relapse after orchidectomy alone [2,3]. Until today no reliable biological parameters exist and clinical parameter are showing a concordance of 65% only in differentiating occult metastasized stages from non-metastasized seminoma [4]. Identification of occult metastasized patients is one of the main goals to prevent toxicity (e.g. cardiovascular and kidney disease, secondary malignancies and decreased fertility) caused by unnecessary adjuvant treatment or diagnostic procedures (additional radiation exposure due to quarterly CT scans) during follow up [5].

Recently, other authors started to examine whether a certain set of micro RNAs (miRNA) might be suitable for discriminating between seminoma bearing patients and healthy persons [6-8]. Expression of miRNAs in testicular germ cell cancer is also known to be associated with the histologic subtype [7] as well as cisplatin resistance [9,10]. Additionally miRNAs are known to be involved in different processes of metastatic spread in other tumours [11]. Among seminoma bearing patients circulating tumour cells are already detected in the peripheral blood [12]. We wondered whether changes in microRNA expression in the peripheral blood might be able to discriminate metastasized (either lymphogen, occult or a combination of both subtypes) from non-metastasized seminoma. We utilized an agnostic approach investigating the whole genome for any kind of small RNA species suitable to discriminate metastatic stage in seminoma employing next generation sequencing (NGS) on peripheral blood samples drawn at the time of the primary tumour’s diagnosis.

## Results

### Characteristics of seminoma groups

The average age at diagnosis was 39.1 (+/- 7.2) years for non-metastasized, higher (45.2 years, +/- 10.8) for lymphogen metastasized and lower (32.1 years, +/- 5.4) for occult metastasized seminoma. Primary tumor size was comparable between lymphogen and occult metastasized seminoma (37.8 mm and 38.6 mm, respectively), but smaller (23.8 mm) in non-metastasized seminoma (Table 1).

**Table 1:** Characteristics of patients, their biopsies and RNA isolates

**#**	**Metastasis detection at time of primary tumor’s diagnosis**	**Age at diagnosis (years)**	**Tumor size (mm)**	**pL**	**pV**	**pT**	**Infiltration rete testis**	**Initial clinical stage**	**Total RNA (μg)**	**RIN**
1	Non metastasized	38	14	0	0	1	n	cSI	7.6	7.8
2		50	22	0	0	1	n	cSI	8.6	8.3
3		31	19	1	0	1	n	cSI	6.4	7.0
4		42	45	0	0	1	y	cSI	3.9	8.0
5		35	19	0	0	1	y	cSI	8.2	7.6
*Mean*		*39.1*	*23.8*						*6.9*	*7.7*
*stdev*		*7.2*	*12.2*						*1.9*	*0.5*
1	Lymphogen metastasized	32	40	0	0	1	n	cSIIb	5.0	8.5
2		50	12	0	0	3	n	cSIIc	5.1	8.8
3		43	50	0	0	1	n	cSIIb	8.1	8.3
4		42	45	1	0	2	y	cSIIb	3.2	9.3
5		61	42	0	0	1	y	cSIIc	3.2	8.2
*Mean*		*45.2*	*37.8*						*4.9*	*8.6*
*stdev*		*10.8*	*14.9*						*2.0*	*0.4*
1	Occult metastasized	33	55	1	0	2	n	cSI	5.0	6.0
2		37	35	0	0	1	y	cSI	4.1	7.4
3		37	30	0	0	1	n	cSI	7.0	7.8
4		31	18	0	0	1	n	cSI	8.1	7.4
5		23	55	0	0	1	n	cSI	10.0	6.4
*Mean*		*32.1*	*38.6*						*6.8*	*7.0*
*stdev*		*5.4*	*16.2*						*2.4*	*0.8*

### RNA isolation

Per 2.5 ml whole blood we isolated about the same amount (mean) of total RNA from seminoma patients without metastasis (7.7 μg, +/-1.9), lymphogen metastasis (4.9 μg, +/-2.0 μg) and occult metastasis (6.8 μg, +/-2.4, Table 1). The RNA integrity number (RIN) ranged between 7.0-8.6 per group. No DNA contamination could be detected in our samples.

### Analysis of small RNA next generation sequencing results

**Figure 1:**
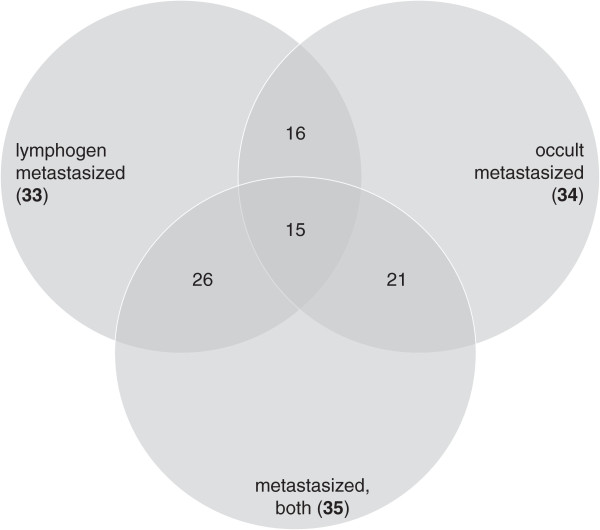
**Venn diagram showing the total number (bold) and overlapping number of significantly associated small RNAs (from Table **3**) to discriminate different metastasis subtypes (lymphogen, occult and the combination of both subtypes) from non-metastasized seminoma.**

The average total number of reads for lymphogen/occult metastasized and non-metastasized seminoma was 1.3x10^7^, 1.2x10^7^ and 1.2x10^7^ and on average 73-76% remained for further analysis after trimming of the reads (Table 2). From these reads 80-84% appeared annotated reads with 7.2-7.8% (1.6-1.7x10^4^) representing annotated small RNAs. Of them 137 small RNAs showed > 50 reads and a two-fold difference to the reference. In univariate analysis we identified 33/34 and 35 small RNA species which significantly discriminated lymphogen/occult metastasized and both metastasis subtypes combined from non-metastasized seminoma, respectively (Table 3). The overlap of small RNAs separating either lymphogen or occult metastasis from non-metastasized seminoma was 47-48% and increased up to 79% when comparing lymphogen and occult small RNAs candidates with the small RNAs based on the combined metastasis subtypes (Figure 1). We finally employed support vector machines which completely separated lymphogen, occult metastasis and the combined metastasis subtypes from non-metastasized seminoma using a combination of two small RNA species only (Table 4). Altogether, 891, 668 and 87 combinations allowed a complete separation of lymphogen (n = 5), occult metastasis (n = 5) and the combined metastasis subtypes (n = 10) from non-metastasized seminoma (n = 5), respectively.

**Table 2:** Descriptive statistics of number of reads before and after trimming and the percentage of annotated small RNAs measured in the peripheral blood of patients suffering from seminoma without metastasis as well as lymphogen and occult metastasized seminoma

**Seminoma metastasis status**	**Total no. of reads**	**Reads after trimming**		**Annotated reads**		**Small RNA tags**		
		**abs.**	**%**	**abs.**	**%**	**abs.**	**abs. annotated**	**%**
***Non-metastasized,*** *n = 5*								
Mean	1.3E + 07	9.7E + 06	76	8.1E + 06	84	2.2E + 05	1.6E + 04	7.2
Stdev	2.0E + 06	1.6E + 06	2.2	1.4E + 06	2.2	5.4E + 04	3.4E + 03	0.7
Min	9.4E + 06	7.1E + 06	72	5.7E + 06	81	1.7E + 05	1.2E + 04	6.5
Max	1.4E + 07	1.1E + 07	78	9.5E + 06	85	3.1E + 05	2.0E + 04	8.2
***Lymphogen metastasized,*** *n = 5*								
Mean	1.2E + 07	7.8E + 06	73	6.3E + 06	80	2.3E + 05	1.6E + 04	7.3
Stdev	6.5E + 06	3.1E + 06	16	2.7E + 06	3.8	8.6E + 04	3.2E + 03	1.5
Min	5.7E + 06	4.8E + 06	45	3.6E + 06	75	1.5E + 05	1.3E + 04	5.6
Max	2.0E + 07	1.2E + 07	86	1.0E + 07	85	3.3E + 05	1.9E + 04	8.5
***Occult metastasized,*** *n = 5*								
Mean	1.2E + 07	9.0E + 06	73	7.3E + 06	82	2.3E + 05	1.7E + 04	7.8
Stdev	4.3E + 06	3.5E + 06	6.6	2.8E + 06	2.5	5.1E + 04	9.4E + 02	1.4
Min	6.7E + 06	4.7E + 06	64	3.8E + 06	79	1.8E + 05	1.6E + 04	5.9
Max	1.9E + 07	1.4E + 07	81	1.1E + 07	85	3.1E + 05	1.8E + 04	10

**Table 3:** Summary of significantly associated small RNAs with metastasis status per group using logistic regression analysis (univariate)

***Non versus metastasized (lymphogen and occult, n = 35)***					***Non versus lymphogen metastasized (n = 33)***					***Non versus occult metastasized (n = 34)***				
**Small RNA**	**p-value**	**Fold-change**	**95% confidence interval**		**Small RNA**	**p-value**	**Fold-change**	**95% confidence interval**		**Small RNA**	**p-value**	**Fold-change**	**95% confidence interval**	
let-7f-1	0.04	5.5	1.3	23.7	let-7f-1	0.03	7.2	1.6	32.4	let-7a-1	0.02	3.1	1.4	7.0
mir-16-1	0.05	2.2	1.1	4.5	let-7f-2	0.04	8.1	1.5	44.6	let-7a-2	0.02	3.1	1.4	7.1
mir-18a	0.05	2.4	1.1	5.2	mir-15a	0.05	5.0	1.3	19.4	let-7a-3	0.02	3.1	1.4	7.0
mir-23a	0.05	2.7	1.1	6.5	mir-16-1	0.001	3.2	2.1	5.0	let-7b	0.01	3.1	1.6	6.1
mir-92a-1	0.008	2.7	1.4	5.1	mir-92a-1	0.007	3.0	1.6	5.5	let-7d	0.006	2.7	1.6	4.6
mir-92a-2	0.008	2.8	1.5	5.2	mir-92a-2	0.007	3.0	1.6	5.6	let-7f-1	0.006	3.8	1.9	7.6
mir-99a	0.01	2.5	1.3	4.6	mir-183	0.001	2.5	1.7	3.6	let-7f-2	0.01	3.8	1.7	8.3
mir-183	0.02	2.0	1.2	3.5	let-7 g	0.03	6.2	1.6	23.0	mir-18a	0.04	2.3	1.2	4.5
let-7 g	0.05	4.5	1.2	17.6	mir-15b	0.02	6.8	1.8	26.2	mir-92a-1	0.03	2.5	1.3	4.8
mir-23b	0.04	2.8	1.1	7.1	mir-23b	0.04	3.3	1.3	8.3	mir-92a-2	0.03	2.5	1.3	4.8
mir-130a	0.001	0.2	0.1	0.4	mir-130a	0.04	0.2	0.1	0.7	mir-99a	0.006	2.5	1.5	4.0
mir-191	0.004	0.4	0.2	0.7	mir-142	0.03	0.5	0.3	0.8	let-7 g	0.03	2.9	1.3	6.4
mir-296	0.01	2.3	1.3	4.0	mir-191	0.03	0.4	0.2	0.8	mir-30b	0.03	2.2	1.2	3.8
mir-378a	0.01	2.4	1.3	4.3	mir-126	0.03	2.0	1.2	3.4	mir-130a	0.03	0.1	0.0	0.5
mir-326	0.02	2.7	1.3	5.5	mir-296	0.01	2.2	1.4	3.6	mir-191	0.01	0.4	0.2	0.7
mir-331	0.01	3.0	1.4	6.3	mir-331	0.02	2.7	1.4	5.3	mir-29c	0.04	2.1	1.1	3.8
mir-339	0.04	2.1	1.1	4.0	mir-425	0.05	3.7	1.2	11.0	mir-296	0.04	2.4	1.2	4.7
mir-425	0.01	3.9	1.6	9.4	mir-451a	0.009	2.2	1.4	3.4	mir-378a	0.006	2.5	1.5	4.2
mir-451a	0.002	2.2	1.5	3.2	mir-92b	0.02	2.2	1.3	3.5	mir-326	0.01	3.4	1.7	6.9
mir-92b	0.02	2.5	1.2	5.0	mir-574	0.005	0.3	0.2	0.6	mir-331	0.03	3.3	1.4	7.9
mir-574	0.0001	0.4	0.2	0.5	mir-4286	0.04	3.3	1.3	8.7	mir-339	0.02	2.6	1.4	4.9
mir-4286	0.02	3.9	1.5	10.5	mir-4454	0.008	2.3	1.4	3.7	mir-425	0.002	4.1	2.2	7.5
mir-4454	0.03	2.7	1.2	5.9	ENST00000516594	0.007	0.4	0.2	0.6	mir-451a	0.01	2.2	1.4	3.5
ENST00000516594	0.02	0.5	0.3	0.8	ENST00000363271	0.007	0.4	0.2	0.6	mir-92b	0.04	2.8	1.2	6.4
ENST00000365160	0.03	0.4	0.2	0.8	ENST00000459091	0.002	0.3	0.2	0.5	mir-574	0.0009	0.4	0.3	0.6
ENST00000363271	0.02	0.5	0.3	0.8	ENST00000516350	0.007	0.4	0.2	0.6	mir-660	0.03	2.4	1.3	4.5
ENST00000459091	0.006	0.4	0.3	0.7	ENST00000517209	0.002	0.4	0.2	0.6	mir-664	0.02	2.1	1.3	3.5
ENST00000516350	0.02	0.5	0.3	0.8	ENST00000516507	0.007	0.4	0.2	0.7	mir-4286	0.02	4.5	1.7	11.8
ENST00000363865	0.02	0.5	0.3	0.8	ENST00000363865	0.007	0.4	0.2	0.6	mir-4454	0.05	3.0	1.2	7.5
ENST00000362808	0.02	0.5	0.3	0.8	ENST00000362808	0.007	0.4	0.2	0.6	ENST00000365160	0.04	0.3	0.1	0.8
ENST00000364409	0.02	0.5	0.3	0.8	ENST00000364409	0.007	0.4	0.2	0.6	ENST00000387347	0.01	3.1	1.6	6.0
ENST00000363745	0.02	0.5	0.3	0.8	ENST00000363745	0.007	0.4	0.2	0.6	ENST00000482884	0.05	2.6	1.2	5.6
ENST00000387347	0.03	3.0	1.3	7.3	ENST00000461337	0.007	0.4	0.2	0.6	ENST00000459949	0.002	2.3	1.6	3.4
ENST00000459949	0.003	2.0	1.4	2.9						ENST00000410361	0.02	3.3	1.4	7.8
ENST00000461337	0.02	0.5	0.3	0.8										

**Table 4:** Summary on combinations of two small RNAs (small RNA1 with small RNA2) which together completely discriminate metastasis subtypes (lymphogen, occult and their combination) from non-metastasized seminoma employing support vector machines

***Non versus metastasized (lymphogen and occult, n = 87)***		***Non versus lymphogen metastasized (n = 87 from 891)***		***Non versus occult metastasized (=87 from 668)***	
**Small RNA1**	**Small RNA2**	**Small RNA1**	**Small RNA2**	**Small RNA1**	**Small RNA2**
let-7f-1	mir-191	let-7a-1	mir-192	let-7a-1	mir-223
let-7f-1	mir-574	let-7a-1	mir-191	let-7a-1	mir-574
let-7f-2	mir-191	let-7a-1	mir-532	let-7a-1	mir-4454
mir-18a	mir-532	let-7a-1	ENST00000516594	let-7a-2	mir-223
mir-19b-1	mir-342	let-7a-1	ENST00000363271	let-7a-2	mir-574
mir-19b-1	ENST00000517209	let-7a-1	ENST00000459091	let-7a-2	mir-4454
mir-19b-2	mir-342	let-7a-1	ENST00000516350	let-7a-3	mir-223
mir-19b-2	ENST00000517209	let-7a-1	ENST00000517209	let-7a-3	mir-574
mir-28	mir-574	let-7a-1	ENST00000516501	let-7a-3	mir-4454
mir-29a	mir-223	let-7a-1	ENST00000516507	let-7b	mir-16-1
mir-29a	mir-574	let-7a-1	ENST00000363865	let-7b	mir-19b-1
mir-93	mir-532	let-7a-1	ENST00000362808	let-7b	mir-19b-2
mir-99a	mir-574	let-7a-1	ENST00000364409	let-7b	mir-22
mir-192	ENST00000459091	let-7a-1	ENST00000363745	let-7b	mir-23a
mir-197	mir-92b	let-7a-1	ENST00000461337	let-7b	mir-24-1
mir-197	mir-4454	let-7a-2	mir-192	let-7b	mir-24-2
mir-183	mir-191	let-7a-2	mir-191	let-7b	mir-25
mir-223	mir-145	let-7a-2	mir-532	let-7b	mir-26a-1
mir-223	mir-423	let-7a-2	ENST00000516594	let-7b	mir-26b
mir-223	mir-664	let-7a-2	ENST00000363271	let-7b	mir-28
mir-223	ENST00000459091	let-7a-2	ENST00000459091	let-7b	mir-29a
mir-30b	mir-574	let-7a-2	ENST00000516350	let-7b	mir-29b-1
mir-125b-1	mir-130a	let-7a-2	ENST00000517209	let-7b	mir-29b-2
mir-130a	mir-142	let-7a-2	ENST00000516501	let-7b	mir-103a-2
mir-130a	mir-125b-2	let-7a-2	ENST00000516507	let-7b	mir-103a-1
mir-130a	mir-150	let-7a-2	ENST00000363865	let-7b	mir-107
mir-130a	mir-186	let-7a-2	ENST00000362808	let-7b	mir-16-2
mir-130a	mir-361	let-7a-2	ENST00000364409	let-7b	mir-192
mir-130a	mir-340	let-7a-2	ENST00000363745	let-7b	mir-197
mir-130a	mir-342	let-7a-2	ENST00000461337	let-7b	mir-181a-2
mir-130a	mir-574	let-7a-3	mir-192	let-7b	mir-182
mir-140	mir-342	let-7a-3	mir-191	let-7b	mir-181a-1
mir-140	mir-532	let-7a-3	mir-532	let-7b	mir-221
mir-142	mir-326	let-7a-3	ENST00000516594	let-7b	mir-223
mir-142	mir-423	let-7a-3	ENST00000363271	let-7b	mir-23b
mir-142	mir-425	let-7a-3	ENST00000459091	let-7b	mir-125b-1
mir-142	mir-451a	let-7a-3	ENST00000516350	let-7b	mir-130a
mir-191	mir-92b	let-7a-3	ENST00000517209	let-7b	mir-140
mir-29c	mir-342	let-7a-3	ENST00000516501	let-7b	mir-142
mir-29c	mir-574	let-7a-3	ENST00000516507	let-7b	mir-191
mir-378a	mir-532	let-7a-3	ENST00000363865	let-7b	mir-125a
mir-378a	mir-574	let-7a-3	ENST00000362808	let-7b	mir-125b-2
mir-342	mir-423	let-7a-3	ENST00000364409	let-7b	mir-126
mir-342	mir-425	let-7a-3	ENST00000363745	let-7b	mir-186
mir-342	mir-3676	let-7a-3	ENST00000461337	let-7b	mir-320a
mir-342	ENST00000387347	let-7b	mir-192	let-7b	mir-106b
mir-342	ENST00000482884	let-7b	mir-191	let-7b	mir-29c
mir-326	mir-532	let-7b	mir-532	let-7b	mir-296
mir-326	mir-574	let-7b	ENST00000516594	let-7b	mir-26a-2
mir-326	ENST00000517209	let-7b	ENST00000363271	let-7b	mir-361
mir-423	mir-574	let-7b	ENST00000459091	let-7b	mir-378a
mir-425	mir-532	let-7b	ENST00000516350	let-7b	mir-340
mir-425	mir-629	let-7b	ENST00000517209	let-7b	mir-328
mir-425	ENST00000517209	let-7b	ENST00000516501	let-7b	mir-342
mir-451a	mir-574	let-7b	ENST00000516507	let-7b	mir-326
mir-484	mir-574	let-7b	ENST00000363865	let-7b	mir-151a
mir-532	mir-92b	let-7b	ENST00000362808	let-7b	mir-331
mir-532	mir-625	let-7b	ENST00000364409	let-7b	mir-324
mir-532	mir-660	let-7b	ENST00000363745	let-7b	mir-339
mir-532	ENST00000482884	let-7b	ENST00000461337	let-7b	mir-345
mir-92b	mir-574	let-7d	mir-192	let-7b	mir-423
mir-574	mir-625	let-7d	mir-191	let-7b	mir-425
mir-574	mir-454	let-7d	mir-532	let-7b	mir-451a
mir-574	mir-664	let-7d	ENST00000516594	let-7b	mir-484
mir-574	mir-4454	let-7d	ENST00000363271	let-7b	mir-505
mir-574	ENST00000516594	let-7d	ENST00000459091	let-7b	mir-532
mir-574	ENST00000363271	let-7d	ENST00000516350	let-7b	mir-574
mir-574	ENST00000459091	let-7d	ENST00000517209	let-7b	mir-652
mir-574	ENST00000516350	let-7d	ENST00000516501	let-7b	mir-766
mir-574	ENST00000516507	let-7d	ENST00000516507	let-7b	mir-744
mir-574	ENST00000363865	let-7d	ENST00000363865	let-7b	mir-1260a
mir-574	ENST00000362808	let-7d	ENST00000362808	let-7b	mir-1280
mir-574	ENST00000364409	let-7d	ENST00000364409	let-7b	mir-1260b
mir-574	ENST00000363745	let-7d	ENST00000363745	let-7b	mir-4286
mir-574	ENST00000387347	let-7d	ENST00000461337	let-7b	mir-3676
mir-574	ENST00000459949	let-7f-1	mir-192	let-7b	mir-4454
mir-574	ENST00000461337	let-7f-1	mir-191	let-7b	ENST00000516572
mir-1260a	ENST00000459091	let-7f-1	mir-532	let-7b	ENST00000516351
mir-3676	ENST00000517209	let-7f-1	ENST00000516594	let-7b	ENST00000516461
mir-4454	ENST00000459091	let-7f-1	ENST00000363271	let-7b	ENST00000516775
mir-4454	ENST00000466665	let-7f-1	ENST00000459091	let-7b	ENST00000516594
ENST00000516572	ENST00000459091	let-7f-1	ENST00000516350	let-7b	ENST00000365160
ENST00000516351	ENST00000459091	let-7f-1	ENST00000517209	let-7b	ENST00000363271
ENST00000459091	ENST00000463508	let-7f-1	ENST00000516501	let-7b	ENST00000459091
ENST00000459091	ENST00000482884	let-7f-1	ENST00000516507	let-7b	ENST00000516350
ENST00000459091	ENST00000488123	let-7f-1	ENST00000363865	let-7b	ENST00000517209
ENST00000517209	ENST00000482884	let-7f-1	ENST00000362808	let-7b	ENST00000516501

From the total number of small RNA combinations per group (shown in parenthesis) only 87 combinations per group are presented.

## Discussion

In our study we aimed to better discriminate metastasized (either lymphogen or occult subtypes or both combined) from non-metastasized seminoma based on small RNA changes examined in the peripheral blood. A whole genome screening on small RNA species was performed employing NGS. We demonstrated each metastasis subtype and its combination to be completely discriminated from non-metastasized seminoma using two small RNAs combined.

MicroRNAs are previously described to be involved in different distant (e.g. migration, angiogenesis and colonization of distant organs) and local (e.g. changes in tumor microenvironment) processes in metastatic cascade. Interestingly, the microRNAs identified in our study are involved in local tumour microenvironmental changes only. For instance, members of the Let-7 family are involved in inflammatory processes as a part of the metastatic cascade. The Let-7 family targets oncogenes such as HMGA2 and KRAS and seem to be a key component in a so called epigenetic switch [13-15]. Cancer associated fibroblasts are involved in tumor formation and progression where miR-15 and miR-16 regulate FGF2 and FGFR1 in prostate cancer [16] and miR-18 in breast cancer [17].

Recently other authors demonstrated that microRNA 371-73 cluster and microRNA 302 discriminated seminoma bearing patients from healthy persons [6-8]. We did the next step and examined the suitability of these microRNA to predict the metastasis status, these microRNA 371-73 cluster and miRNA 302 was detectable in our analysis, but appeared not significantly associated with the metastasis status as it is true for protein tumour markers (e.g. HCG) as well.

The complete separation of metastasis from non-metastasized seminoma using a combination of two small RNA species points to the significant diagnostic potential of these biological marker which is in line with previous examinations on the transcriptional level showing the superiority of molecular marker over epidemiological or clinical-histological parameter [18,19]. After validation of these results on a larger group, these findings could help for therapy decision making in favour of adjuvant chemotherapy or surveillance based on miRNA expression changes predicting metastasis spread, even if metastasis spreads are not visible using a CT scan.

Interesting, the discrimination in our study occurred irrespective of the metastasis subtype and with the same microRNAs in up to 79%. This was expected, since we recently demonstrated lymphogen and occult metastasized seminoma to be indistinguishable on the transcriptional and post-transcriptional level (revised version submitted).

Noteworthy, these changes were examined in the peripheral blood of seminoma patients taken at the time of the primary tumour’s diagnosis. It can be hypothesized that our measurements are linked to seminoma tumour cells circulating in the peripheral blood.

Our study has certain weaknesses such as the low number of cases examined (total n = 15). However, the molecular biological methodology applied provides a deepness, which under financial considerations does not allow large scale studies. Hence, this study provides hints towards certain small RNA species comprising a significant diagnostic potential for prediction of seminoma metastasis based on examinations in the peripheral blood, but certainly these candidate small RNA species have to be examined on a larger independent group for validation purposes. The validation should also include haematogenous metastasized seminoma (clinical Stage III), not included in this study, to prove the usability in this tumor stage as well. Furthermore, we did not adjust the p-values for multiple comparisons, because of the explorative character of this study and the low sample size. Based on experiences with previous work we expect about 10-20% of our candidates being false positives/negatives [20]. However, this study represents a screening approach. About 100 most promising candidate genes will be considered for validation in a following study using another group and another methodology (qRT-PCR).

## Conclusion

Metastasized (either lymphogen or occult) seminoma can be completely discriminated from non-metastasized seminoma with a combination of two small RNAs measured in the peripheral blood.

## Methods

### Patient selection

Non-metastasized seminoma (n = 5) received no adjuvant treatment and were free of relapse/progress for at least two years of follow up. Occult metastasized patients (n = 5) presented without visible metastasis at primary staging, received no adjuvant treatment, and developed retroperitoneal tumour progress during the follow up of 12 month. For patients with detectable metastasis at primary staging (n = 5) we focused on clinical stage IIb and IIc, to include lymphogenic metastatic spread only and to provide a high level of diagnostic accuracy (avoiding doubtful lymph nodes). Lymphogen metastasis and non-metastasized patients were matched with occult metastasized patients considering demographic and histological parameters where applicable as well as quality criteria for isolated RNA (Table 1). All patients included in this study were treated between 2008 and 2010 in one testis cancer centre. These 15 patients were selected out of about 300 seminoma patients, according to the criteria mentioned above.

### Histological examination

All testicular tumours (n = 15) were examined by an experienced pathologist for histological and TNM classification (Table 1) and pure seminoma was diagnosed in all cases.

### Blood sample taking

Whole blood samples (2.5 ml) were taken intraoperative from cubital vein directly into the PAXgene Blood RNA system (BD Diagnostics, PreAnalytiX GmbH, Hombrechtikon, Switzerland). The tube was gently inverted (10 times), settled at room temperature overnight and stored at -20° until use. The Ethical Review Committee of the Medical Association Hamburg approved the study and all human samples were obtained with written informed consent.

### RNA Isolation

After thawing the PAXGene tubes, and washing and centrifugation of the samples the cells became lysed (Proteinase K) followed by adding Lysis/Binding Solution taken from the mirVana Kit (Life Technologies, Darmstadt, Germany). With the mirVana kit total RNA including small RNA species was isolated by combining a Phenol-Chloroform RNA precipitation with further processing using a silica membrane. After several washing procedures DNA residuals became digested on the membrane (RNAse free DNAse Set, Qiagen, Hilden, Germany), washed, RNA was eluted in a collection tube and frozen at -80°C. Quality and quantity of isolated total RNA were measured spectrophotometrically (NanoDrop, PeqLab Biotechnology, Erlangen, Germany). RNA integrity was assessed by the 2100 Agilent Bioanalyser (Life Science Group, Penzberg, Germany) and DNA contamination was controlled by conventional PCR using actin primer.

For analysis, we used only RNA specimens with a ratio of A260/A280 ≥ 2.0 (Nanodrop) and RNA integrity number (RIN) between 6.0-9.3 for small RNA Next Generation Sequencing (IMGM Laboratories, Martinsried, Germany/CeGat, Tübingen, Germany). For RNA samples with RIN below 7.0 or questionable 28/18S bands (indications of RNA degradation) we performed additional checks to exclude the presence of RNAses and true RNA-degradation.

### Small RNA next generation sequencing and data analysis

We performed a genome wide small RNA sequencing using the SOLiD5500xl Next Generation Sequencing Technology (Life Technologies, Penzberg, Germany). In brief, total RNA was purified (PureLink microRNA isolation Kit), enriched small RNAs were ligated to SOLiD adaptors, reverse transcribed (SOLiD RT primer and ArrayScript RT), cDNA was purified (MinElute PCR purification Kit, Qiagen), a cut-off size of 60-80 nt was selected (Novex pre-cast gel products, invitrogen), cDNA was in-gel amplified and samples were barcoded using SOLiD 3′Barcode primer at the same time. Amplified cDNA was purified (PureLink PCR Micro Kit, Life Technologies) and used in emulsion PCR (SOLiD EZ Bead). Thereafter, the emulsion was broken to recover enriched beads and the so-called di-base probes were used by the SOLiD system in the sequencing-by-ligation procedure. Beside the SOLiD5500xl inherent software (LifeScope) used for image and signal processing, software CLC Genomics Workbench 5.1 (CLC bio) was used for clustering, counting, and annotation of all generated 75 bp reads. After discarding reads without adaptor sequence and being shorter than 15 bp (trimming) reads were assigned to known microRNAs (Sanger miRBase release 18, http://www.mirbase.org/) and known non-coding RNAs (ensembl database Homo_sapiens.GRCh37.67.ncrna.fa, http://www.ensembl.org). Small RNAs showing a significant and ≥ 2-fold differences in gene expression among groups and at least 50 reads were further analysed on their ability to separate both groups using univariate logistic regression analysis [21] and combinations of two small RNAs were examined employing support vector machines (linear Kermel).

## Abbreviations

bp: Base pair; cDNA: Copy deoxyribonucleic acids; cS: clincal stage; CT scan: Computed tomography; DNA: Deoxyribonucleic acids; miRNA: micro ribonucleic acid; NGS: Next generation sequencing; qRT-PCR: Quantitative real time polymerase chain reaction; RNA: Ribonucleic acid; RIN: RNA integrity number; TNM-Classicfication: Tumor nodus metastasis classification.

## Competing interests

The German Ministry of Defence supported this work. All authors declare that they have no competing interests.

## Authors’ contributions

CR has developed the study design, acquisition and interpretation of data and drafted the manuscript, DD has substantially contributed in the acquisition of data, MP and H-US have substantially contributed in the study design, interpretation of data and in revising the manuscript, WW and CM have made substantial contributions to acquisition of data and in revising the manuscript, BM-M has performed the statisitcal analysis of data and revised the manuscript, VM has made substantial contributions to technical procedures, data analysis and revised the manuscript, MA has developed the study design, supervised all technical/laboratory procedures and quality checks, analysis and interpretation of data and drafted the manuscript. All authors read and approved the final manuscript.

## References

[B1] Ruf CG Isbarn H Wagner W Fisch M Matthies C Dieckmann KP Changes in epidemiologic features of testicular germ cell cancer: age at diagnosis and relative frequency of seminoma are constantly and significantly increasing() Urol Oncol 2014 32 1 33 e1-6. doi:10.1016/j.urolonc.2012.12.002. Epub 2013 Feb 6 23395239 10.1016/j.urolonc.2012.12.002

[B2] Krege S Beyer J Souchon R Albers P Albrecht W Algaba F Bamberg M Bodrogi I Bokemeyer C Cavallin-Stahl E Classen J Clemm C Cohn-Cedermark G Culine S Daugaard G De Mulder PH De Santis M de Wit M de Wit R Derigs HG Dieckmann KP Dieing A Droz JP Fenner M Fizazi K Flechon A Fosså SD del Muro XG Gauler T Geczi L European consensus conference on diagnosis and treatment of germ cell cancer: a report of the second meeting of the European Germ Cell Cancer Consensus group (EGCCCG): part I Eur Urol 2008 53 478 496 10.1016/j.eururo.2007.12.024 18191324

[B3] Albers P Albrecht W Algaba F Bokemeyer C Cohn-Cedermark G Fizazi K Horwich A Laguna MP [EAU guidelines on testicular cancer: 2011 update. European Association of Urology] Actas Urol Esp 2012 36 127 145 22188753 10.1016/j.acuro.2011.06.017

[B4] Ruf CG Khalili-Harbi N Sachs S Isbarn H Wagner W Matthies C Meineke V Fisch M Chun FK Abend M The search for biomarkers of metastatic seminoma J Urol 2013 190 3 1046 1051 doi:10.1016/j.juro.2013.04.022. Epub 2013 Apr 10 10.1016/j.juro.2013.04.022 23583226

[B5] Kollmannsberger C Tyldesley S Moore C Chi KN Murray N Daneshmand S Black P Duncan G Hayes-Lattin B Nichols C Evolution in management of testicular seminoma: population-based outcomes with selective utilization of active therapies Ann Oncol 2011 22 808 814 10.1093/annonc/mdq466 20926549

[B6] Dieckmann KP Spiekermann M Balks T Flor I Loning T Bullerdiek J Belge G MicroRNAs miR-371-3 in serum as diagnostic tools in the management of testicular germ cell tumours Br J Cancer 2012 107 1754 1760 10.1038/bjc.2012.469 23059743 PMC3493876

[B7] Gillis AJ Stoop HJ Hersmus R Oosterhuis JW Sun Y Chen C Guenther S Sherlock J Veltman I Baeten J van der Spek PJ de Alarcon P Looijenga LH High-throughput microRNAome analysis in human germ cell tumours J Pathol 2007 213 319 328 10.1002/path.2230 17893849

[B8] Palmer RD Murray MJ Saini HK van Dongen S Abreu-Goodger C Muralidhar B Pett MR Thornton CM Nicholson JC Enright AJ Coleman N Children's Cancer and Leukaemia Group Malignant germ cell tumors display common microRNA profiles resulting in global changes in expression of messenger RNA targets Cancer Res 2010 70 2911 2923 10.1158/0008-5472.CAN-09-3301 20332240 PMC3000593

[B9] Port M Glaesener S Ruf C Riecke A Bokemeyer C Meineke V Honecker F Abend M Micro-RNA expression in cisplatin resistant germ cell tumor cell lines Mol Cancer 2011 10 52 10.1186/1476-4598-10-52 21575166 PMC3120796

[B10] Liu X Yu J Jiang L Wang A Shi F Ye H Zhou X MicroRNA-222 regulates cell invasion by targeting matrix metalloproteinase 1 (MMP1) and manganese superoxide dismutase 2 (SOD2) in tongue squamous cell carcinoma cell lines Cancer Genomics Proteomics 2009 6 131 139 19487542 PMC2890246

[B11] Profumo V Gandellini P MicroRNAs: cobblestones on the road to cancer metastasis Crit Rev Oncog 2013 18 341 355 10.1615/CritRevOncog.2013007182 23614620

[B12] Ruf CG Nastaly P Becker P Isbarn H Honecker F Pantel K Riethdorf S Hoeppner D Fisch M Wagner W Ahyai S Circulating tumor cells can be detected in patients with testicular germ cell tumors J Urol 2013 189 e289

[B13] Mayr C Hemann MT Bartel DP Disrupting the pairing between let-7 and Hmga2 enhances oncogenic transformation Science 2007 315 1576 1579 10.1126/science.1137999 17322030 PMC2556962

[B14] Johnson SM Grosshans H Shingara J Byrom M Jarvis R Cheng A Labourier E Reinert KL Brown D Slack FJ RAS is regulated by the let-7 microRNA family Cell 2005 120 635 647 10.1016/j.cell.2005.01.014 15766527

[B15] Iliopoulos D Hirsch HA Struhl K An epigenetic switch involving NF-kappaB, Lin28, Let-7 MicroRNA, and IL6 links inflammation to cell transformation Cell 2009 139 693 706 10.1016/j.cell.2009.10.014 19878981 PMC2783826

[B16] Musumeci M Coppola V Addario A Patrizii M Maugeri-Sacca M Memeo L Colarossi C Francescangeli F Biffoni M Collura D Giacobbe A D'Urso L Falchi M Venneri MA Muto G De Maria R Bonci D Control of tumor and microenvironment cross-talk by miR-15a and miR-16 in prostate cancer Oncogene 2011 30 4231 4242 10.1038/onc.2011.140 21532615

[B17] Yu Z Willmarth NE Zhou J Katiyar S Wang M Liu Y McCue PA Quong AA Lisanti MP Pestell RG microRNA 17/20 inhibits cellular invasion and tumor metastasis in breast cancer by heterotypic signaling Proc Natl Acad Sci U S A 2010 107 8231 8236 10.1073/pnas.1002080107 20406904 PMC2889540

[B18] Ruf CG Linbecker M Port M Riecke A Schmelz HU Wagner W Meineke V Abend M Predicting metastasized seminoma using gene expression BJU Int 2012 110 E14 E20 10.1111/j.1464-410X.2011.10778.x 22243760

[B19] Port M Wang Y Schmelz HU Pottek T Meineke V Ruf C Abend M A gene signature of primary tumor identifies metastasized seminoma Urol Oncol 2011 29 764 773 10.1016/j.urolonc.2009.08.008 19945308

[B20] Port M Seidl C Ruf CG Riecke A Meineke V Abend M Reliable and sample saving gene expression analysis approach for diagnostic tool development Health Phys 2012 103 159 168 22951474 10.1097/HP.0b013e31824ac318

[B21] Baggerly KA Deng L Morris JS Aldaz CM Overdispersed logistic regression for SAGE: modelling multiple groups and covariates BMC Bioinform 2004 5 144 10.1186/1471-2105-5-144 PMC524524 15469612

